# Rationally co-targeting divergent pathways in *KRAS* wild-type colorectal cancers by CANscript technology reveals tumor dependence on Notch and Erbb2

**DOI:** 10.1038/s41598-017-01566-x

**Published:** 2017-05-04

**Authors:** Nilesh Brijwani, Misti Jain, Muthu Dhandapani, Farrah Zahed, Pragnashree Mukhopadhyay, Manjusha Biswas, Deepak Khatri, Vinod D. Radhakrishna, Biswanath Majumder, Padhma Radhakrishnan, Saravanan Thiyagarajan

**Affiliations:** 1 Division of Molecular Profiling, Mitra Biotech, Bangalore, Karnataka, 560099 India; 2 Symbiosis International University (SIU), Lavale, Mulshi Taluka, Pune, Maharashtra 412115 India; 3 Division of Cancer Biology, Mitra Biotech, Bangalore, Karnataka 560099 India; 4 Division of Molecular Pathology, Mitra Biotech, Bangalore, Karnataka 560099 India; 5 Division of Oncology Pharmacology, Mitra Biotech, Bangalore, Karnataka 560099 India; 6 Division of Cancer Biology, Mitra Biotech Inc., Woburn, 01801 MA USA

## Abstract

*KRAS* mutation status can distinguish between metastatic colorectal carcinoma (mCRC) patients who may benefit from therapies that target the epidermal growth factor receptor (EGFR), such as cetuximab. However, patients whose tumors harbor mutant *KRAS* (codons 12/13, 61 and 146) are often excluded from EGFR-targeted regimens, while other patients with wild type *KRAS* will sometimes respond favorably to these same drugs. These conflicting observations suggest that a more robust approach to individualize therapy may enable greater frequency of positive clinical outcome for mCRC patients. Here, we utilized alive tumor tissues in *ex-vivo* platform termed CANscript, which preserves the native tumor heterogeneity, in order to interrogate the antitumor effects of EGFR-targeted drugs in mCRC (*n* = 40). We demonstrated that, irrespective of *KRAS* status, cetuximab did not induce an antitumor response in a majority of patient tumors. In the subset of non-responsive tumors, data showed that expression levels of EGFR ligands contributed to a mechanism of resistance. Transcriptomic and phosphoproteomic profiling revealed deregulation of multiple pathways, significantly the Notch and Erbb2. Targeting these nodes concurrently resulted in antitumor efficacy in a majority of cetuximab-resistant tumors. These findings highlight the importance of integrating molecular profile and functional testing tools for optimization of alternate strategies in resistant population.

## Introduction

Colorectal cancer (CRC) is the third most commonly diagnosed cancer worldwide with a 5-year survival rate of less than 10%^1^. An important molecular target implicated in disease progression is Epidermal Growth Factor Receptor (EGFR) signaling, which after ligand binding triggers two main pathways: the RAS-RAF-MAPK cascade leading to cell proliferation, survival, invasion and metastasis; and the PI3K-PTEN-AKT pathway which controls cell survival, motility and neo-angiogenesis^2^. Notably, EGFR is overexpressed in 60–80% of colorectal tumors^3^. Current chemotherapeutic options include 5FU + leucovorin, XELOX, XELIRI, FOLFOX and FOLFIRI which are combinations of capecitabine, 5-fluorouracil, leucovorin and oxaliplatin or irinotecan. Two classes of anti-EGFR monoclonal antibodies (mAbs) are at present prescribed in combination with conventional chemotherapy for the treatment of CRC. However the underlying problem of using cetuximab (a chimeric-IgG1mAb) is that it has only 8.8% efficacy when used in monotherapy, and 22.9% when used in combination therapy for refractory cases^4^. Further compounding the problem is the fact that cetuximab treatment is often accompanied by *de novo* and acquired resistance in metastatic CRC (mCRC) tumors^5^.

Although EGFR overexpression, gene copy number variation and mutational status are widely used for treatment selection in lung tumors, these approaches have demonstrated very limited predictive value for anti-EGFR therapy in CRC^6–9^. This explains why a large subset (~80%) of CRC does not respond to monoclonal antibodies such as cetuximab and panitumumab. In a vast majority of the tumors, multiple defects in the oncogenic RAS pathway trigger the bypass routes of the EGFR signaling such as ligand independent activation, that also to some extent; imply the conflicting response to monoclonal antibodies^10, 11^. The RAS protein is a critical downstream component of EGFR signaling pathway and is highly associated with diverse aspects of colon tumorigenesis such as uncontrolled proliferation, differentiation and deregulated apoptosis^12^. High KRAS activity leads to constitutive activation of the RAS/RAF signaling complex accompanied by elevated ERK activity^13^. This oncogenic addiction is independent of EGFR activation downstream from ligand binding^14^.

While the presence of *KRAS* mutations has been clinically correlated with the lack of response to cetuximab, the absence of mutations does not necessarily signify a favorable outcome. In fact, only 10–40% of CRC patients with wild-type (wt) *KRAS* respond to cetuximab therapy^15^. Furthermore, patients with wt *KRAS* prospectively develop resistance to targeted EGFR blockade after initiation of therapy^16^. Such an aberrant response profile could be attributed to a variety of factors at the genetic, epigenetic and functional levels like *BRAF, PIK3CA* and EGFR ligands namely Amphiregulin/Epiregulin (*AREG/EREG*)^17–21^. Additionally, abnormalities in other pathways including Erbb2, MET, FGFR1, PDGFR, IGF2, NTRK1 and MEK1 can also circumvent EGFR signaling^22–25^. Therefore, in addition to biomarker based screening, functional assay based stratification of patient tumors for predicting response to anti-EGFR therapy opens further scope for informed treatment outcome in a personalized setting^26^.

In this study, we employed an *ex-vivo* tumor explant model (CANscript) combined with guided molecular profiling strategies to elucidate the biology of response and resistance to cetuximab in mCRC^27^. Our findings suggest that in the absence of any reliable predictive response biomarkers, functional evaluation of tumors in coordination with intrinsic genetic and proteomic profiling could mechanistically help in rational targeting of functionally perturbed cascade(s).

## Results

### Metastatic CRC patient tumors harboring non-mutated *KRAS* (12/13, 61 and 146 codons) exhibit divergent dependence on EGFR axis

**Figure 1:**
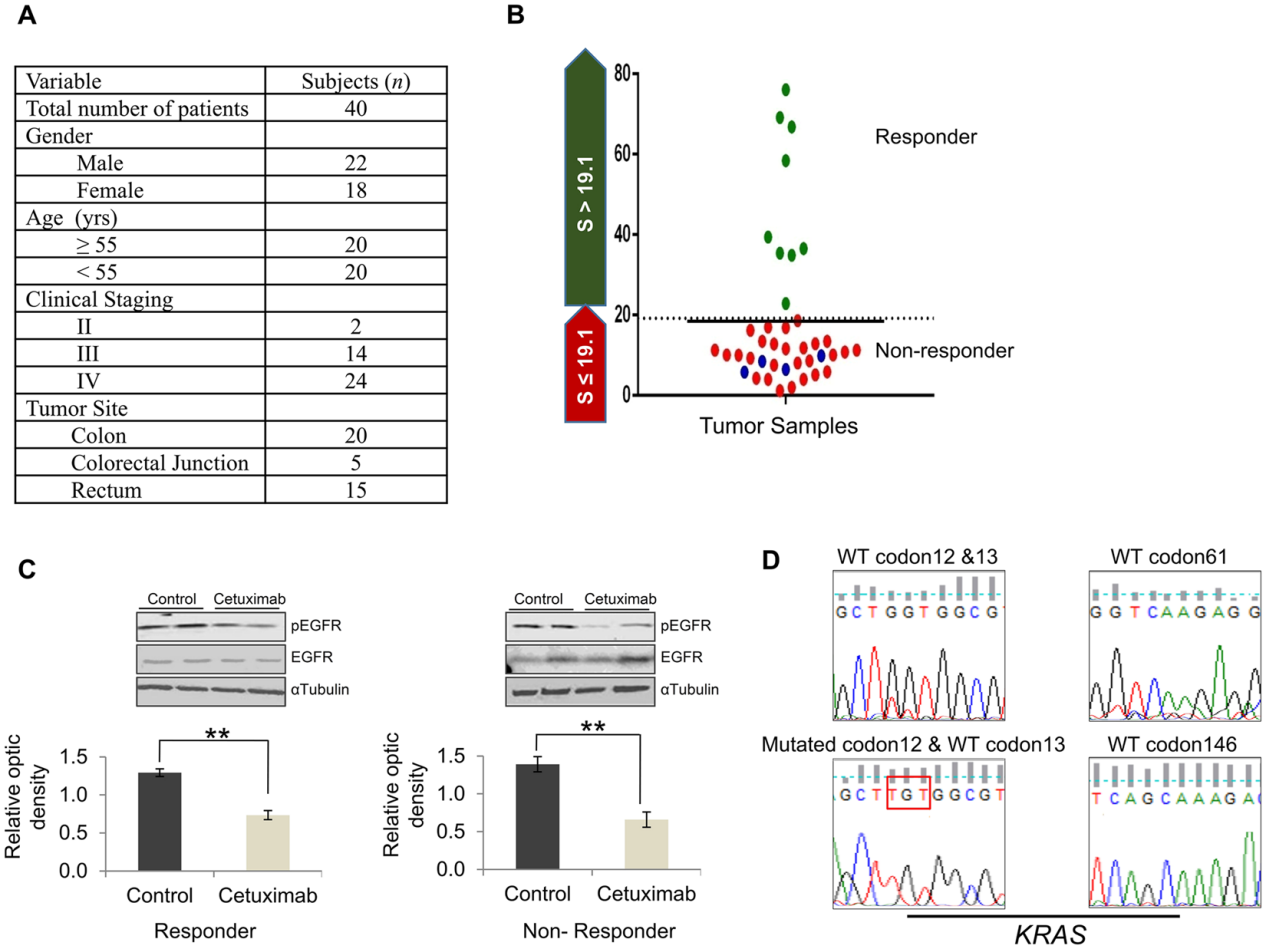
Patient derived CRC explants with wt *KRAS* exhibit differential sensitivity to anti-EGFR agent. (**A**) Demographic details of mCRC patients enrolled for the study. (**B**) Scatter plot depicting S-Score for responders (green) and non-responders (red, *KRAS* wild-type and blue, *KRAS* mutated) to cetuximab (*n* = 40). Samples with S-Score greater than 19.1 were categorized as responders. (**C**) Total protein was isolated from the responder (left) and non-responder (right) tumor samples to cetuximab, separated using SDS-PAGE, blotted on PVDF membrane and incubated with antibody against phospho-EGFR (175kDa). Membranes were re-probed with total EGFR and αTubulin (loading control) respectively. Densitometric analysis for pEGFR was carried out and plotted after normalizing against the loading control. **P < 0.01 was calculated using ANOVA. (**D**) Representative chromatograms depicting wt *KRAS* (codon 12/13, 61 and 146) and mutated *KRAS* (codon 12; red box).

We analyzed forty clinically confirmed distinct CRC patient tumors to ascertain response to cetuximab using the personalized tumor explant culture system termed CANscript as described previously (Fig. 1A)^27^. The platform contextually integrates the explant culture with a machine learning algorithm to better predict clinical outcomes. Treatment efficacy was evaluated by assessing the changes in tumor cell viability (CCK8), morphology (H&E), proliferation (Ki-67) and induction of apoptosis (cleaved caspase-3) after treating tumor explants with cetuximab. Inputs from these parameters were integrated into a mathematical algorithm (**S**VMpAUC)^26^ to generate a single score (S-Score). Our study demonstrates that S-Score^26^ can segregate patients into populations of responders (a value of >19.1) and non-responders to therapy with high positive predictive value (Fig. 1B). Accordingly, tumors that were segregated as responders to cetuximab (9/40, ~22%) showed a significant reduction in viability/tumor content or proliferation and a concomitant increase in activated caspase-3 post-treatment, compared with the vehicle control (Supplementary Fig. S1A–D). In contrast, most tumors (31/40, ~78%), when treated with cetuximab, did not exhibit significant changes in viability, proliferation and apoptosis; they were categorized as non-responders. These findings suggest a differential response pattern consistent with the outcome documented in the clinical setting where CRC patient derived tumors with *KRAS* genes that are non-mutated in codons 12/13, 61 and 146 do not always respond to anti-EGFR agents (Supplementary Fig. S1A–D)^15^. Western blot analysis revealed a decrease in EGFR phosphorylation both in responder and non-responder tumor samples in the drug treatment arms compared with the control, confirming the pharmacodynamic effect as expected in the explant setting (Fig. 1C). Conversely, the total EGFR levels with respect to the loading control, αTubulin remained unaltered in both responder and non-responder groups. Further, to delineate the underlying mechanism(s) of non-response observed in the subset of tumors (31/40, ~78%), we evaluated the mutation status of *KRAS* genes that have a clinically validated link to non-response. Consistent with previous findings, the CRC tumors harboring G12V/C *KRAS* mutations (10% of these tumors) did not show any response to cetuximab treatment *ex-vivo* (Fig. 1D). Despite having non-mutated *KRAS* codons at these positions, a significant percentage (27/36, 75%) of patient tumors were found to be resistant to cetuximab. These observations are in line with published findings that highlight the limited utility of biomarker based segregation of patients with regards to anti-EGFR therapy response^15, 16^.

### Molecular profiling reveals multiple genetic and proteomic deregulations in CRC tumors non-responsive to anti-EGFR therapy

**Figure 2:**
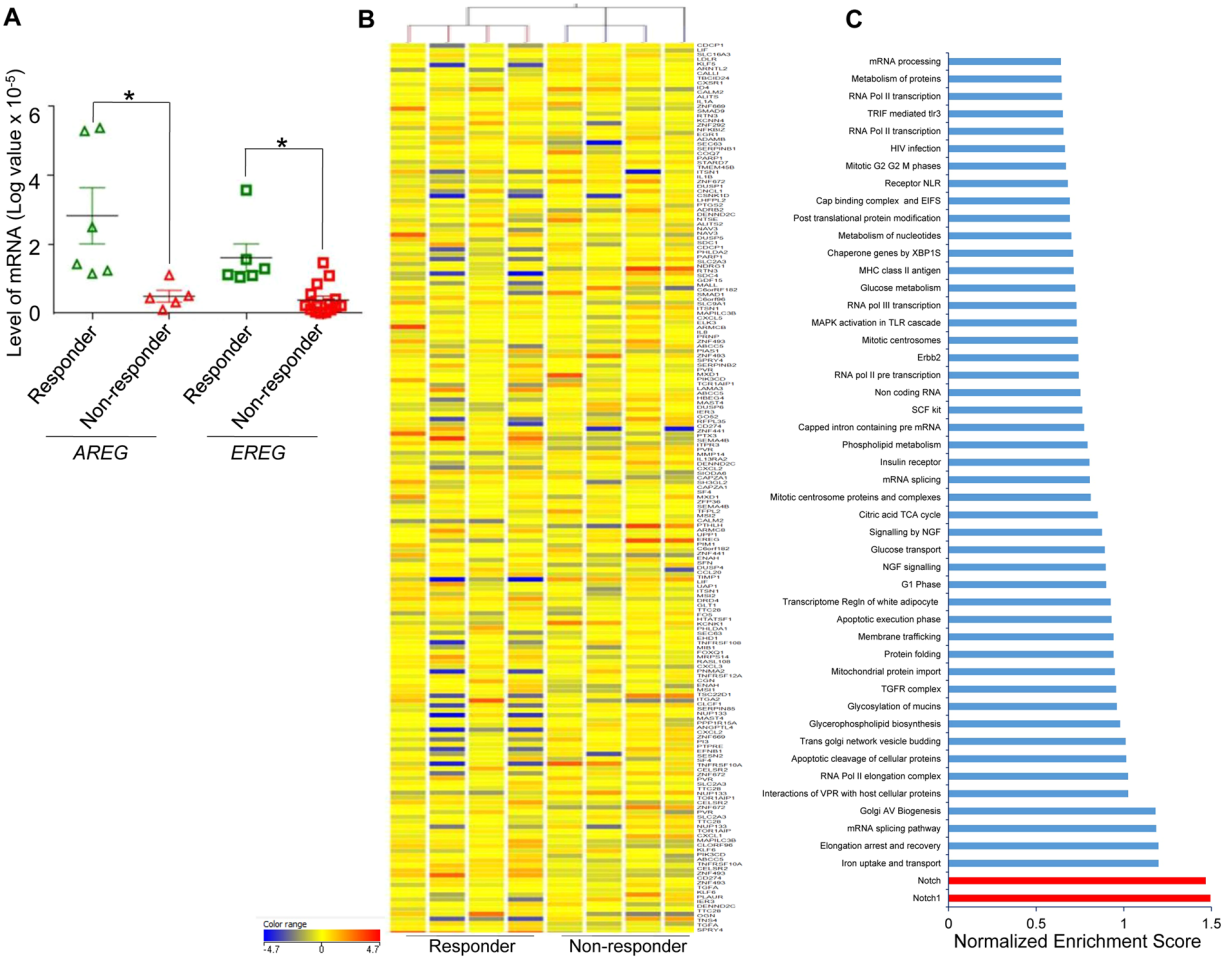
Deregulation of Erbb2 and Notch pathways in CRC tumors with wt *KRAS* but insensitive to anti-EGFR. (**A**) Total RNA isolated from tumors was subjected to qRT-PCR for *AREG* and *EREG*. Expression levels (log transformed values) were calculated and plotted (mean of triplicates ± SE) for *AREG* (triangles) and *EREG* (squares). Samples sensitive to cetuximab are depicted in green and samples insensitive to cetuximab in red. Significance (*P < 0.05, *n* = 27) was calculated by student’s paired t-test. (**B**) Unsupervised heat map clustering for responsive and non-responsive cohort. (*n* = 8). Scale (−4.7 to 4.7) for relative expression levels of different genes is depicted. (**C**) GSEA performed on microarray data from the tumor tissues non-responsive to cetuximab using the reactome database. Fifty gene sets/pathways including Notch (red) and Erbb2 pathways that exhibited significant normalized enrichment score were ranked and plotted.

To study potential molecular mechanism of resistance to cetuximab in the subset of tumors that were wt *KRAS*, we performed comprehensive profiling at the transcriptional level. Independently, we also evaluated the expression of two clinically relevant EGFR ligands, *AREG* and *EREG*, by quantitative real-time (qRT-PCR), as their decreased expression in some cases indicates resistance to anti-EGFR therapy^28^. About 20% of the non-responder tumors (6/27) that were wt *KRAS* at codons 12/13, 61 and 146 expressed very low levels of these ligands (Fig. 2A). We hypothesize that in these tumors, this phenomenon could potentially contribute to the observed lack of response to cetuximab. To further delineate the mechanism of resistance, global transcriptomic profiling of the non-responder and responder tumors was carried out to identify key deregulated pathways or targets that could impact response to anti-EGFR therapy. An unsupervised hierarchical global heat map revealed transcriptionally distinct clusters for both responder and non-responder populations (Fig. 2B). Previous studies have implicated a putative *KRAS* gene signature and its poor score akin to activating *KRAS* mutations and attributing non-response to cetuximab^29, 30^. The mutational analysis of all samples that were non-mutated at specific *KRAS* codons further confirmed the presence of wt *PIK3CA* (exon 9 and 20) and *BRAF* (codon 600) (Supplementary Fig. S2A). Further, analysis of this data in our study revealed that the *KRAS* gene signature was largely similar in the cetuximab sensitive and insensitive CRC tumors (Supplementary Fig. S2B). These findings prompted us to evaluate the enrichment status of additional pathways perceivably, as a consequence of other oncogenic addiction. For this purpose, we utilized gene set enrichment analysis (GSEA) using the c2 gene set database (Reactome) which contains peer reviewed functional pathway data sets^31, 32^. This identified a 116 gene set including pathways involved in cellular metabolism, cell cycle and nutrient uptake. These pathways were predominantly upregulated in non-responder tumors compared with the responders. Notably, the genes related to the Notch pathway which play an important role in colon tumorigenesis, were significantly deregulated in the baseline non-responder tumors (Fig. 2C). Another important protein reported to display aberrant expression in clinically aggressive and anti-EGFR resistant CRC tumors is Erbb2. Using the GSEA platform we identified Erbb2 signaling within the top 20 significantly enriched pathways (Fig. 2C).

**Figure 3:**
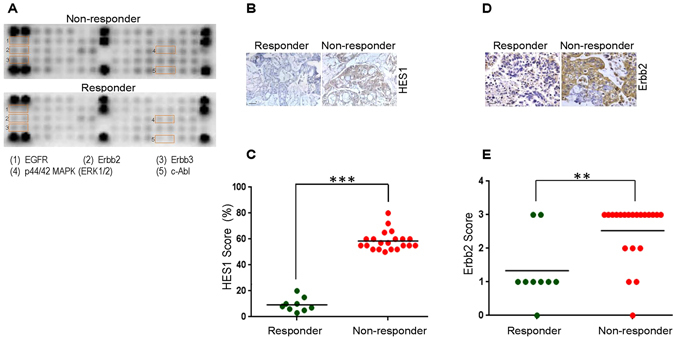
Validation of potential targets identified by transcriptomic analysis in the cohort non-responsive to cetuximab. (**A**) RPPA profiling for multiple targets was performed using total proteins extracted from cetuximab-sensitive (bottom) and cetuximab-resistant (top) tumors at baseline. Spots are in duplicate and each pair corresponds to a specific RTK or signaling node as defined in the array map. The orange insert represents EGFR and associated pathways deregulated in non-responders relative to responders. (**B**) To evaluate the baseline expression of pathway specific markers in responder (left column) and non-responder (right column) tumor samples from mCRC, the formalin fixed and paraffin embedded sections were probed with antibody against HES1. Representative images were taken at 200X magnification. (**C)** Scatter plot depicting HES1 levels in the responder (*n* = 9) and non-responder (*n* = 21) population. HES1 scoring was performed using IHC on a scale of 0 to 100% in each of the tumor sections and significance (***P < 0.001, *n* = 27) was calculated using student’s unpaired t-test. (**D)** To evaluate the baseline expression of Erbb2 in responder (left column) and non-responder (right column) tumor samples from mCRC, formalin fixed and paraffin embedded sections were probed with primary and secondary antibody as indicated. Representative images were taken at 200X magnification. (**E)** Scatter plot depicting Erbb2 levels in the responder (*n* = 9) and non-responder (*n* = 21) population. Erbb2 scoring was performed on a scale of 0 to 3+ using IHC and significance (**P < 0.01, *n* = 27) was calculated using student’s paired t-test.

Next, we sought to elucidate the active status of nodal signaling mechanisms potentially involved in resistance to anti-EGFR therapy. Therefore, we profiled these CRC tumors using whole biopsied tissues at baseline for key phosphoproteins implicated in colon tumorigenesis and survival. Reverse phase phosphoproteomic array (RPPA) analysis profiled 39 total receptors tyrosine kinases (RTKs) including their nodal proteins using a single array platform. Proteins representing the EGFR family, particularly Erbb1, Erbb2 and Erbb3, showed relatively higher level of activation in non-responder tumors compared with their cetuximab-sensitive counterparts (Fig. 3A and Supplementary Fig. S2C). We also found increased levels of c-Abl (a non-RTK) in non-responder tumors which is known to play a role in colon tumorigenesis via Notch signaling, implying the mechanistic crosstalk between Erbb2 and Notch signaling in these tumors^9^. We further ascertained the unequivocal presence of Erbb2 and Notch status in a subset of non-responder tumors by immunohistochemical (IHC) quantification. In the Notch deregulated subset, we observed a high level of expression of the hairy and enhancer of split-1 (HES1) protein (a direct target of the Notch signaling cascade) in most tumor samples tested, suggesting the existence of differentially addicted pathways in these tumors (Fig. 3B and C). A similar pattern was observed for Erbb2 (Fig. 3D and E). Collectively, these data indicate there are diverse mechanisms which might orchestrate the lack of antitumor effect following treatment with cetuximab in clinical CRC samples harboring wild type *KRAS* gene (codons 12/13, 61 and 146).

### Co-targeting Notch and Erbb2 pathways induces a synergistic antitumor effect in CRC tumors insensitive to cetuximab

**Figure 4:**
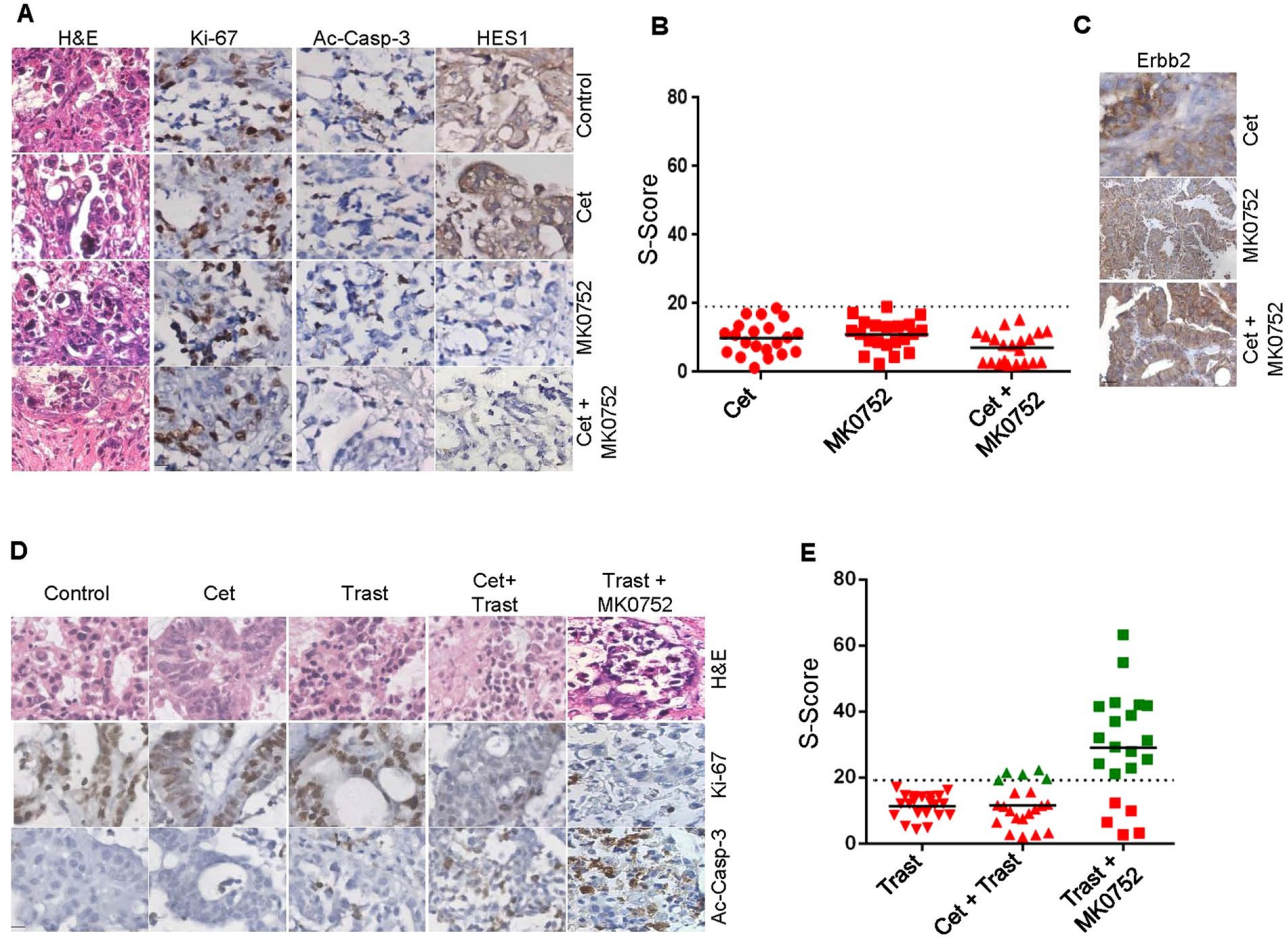
Targeted inhibition of Erbb2 and EGFR elicits antitumor effect in a subset of anti-EGFR insensitive tumors. (**A**) Following three days of culture, tumor tissues in triplicates were formalin fixed and paraffin embedded. Tumor sections treated with the vehicle control (first row), cetuximab (second row), MK0752 (third row), and combination (cetuximab + MK0752) (fourth row) were stained for H&E (first column) and selectively probed with antibodies against Ki-67 (second column), active caspase-3 (third column) and HES1 (fourth column) as indicated. Representative images were taken at 200X magnification. (**B**) Scatter plot depicting response prediction based on S-Score to cetuximab (circles), MK0752 (squares) and cetuximab + MK0752 (triangles) in multiple samples (*n* = 21). Samples with S-Score lesser than or equal to 19.1 were categorized as non-responders. (**C**) To evaluate expression of pathway specific marker, Erbb2 in tumor samples refractory to cetuximab (first row), MK0752 (second row) and cetuximab + MK0752 (third row) were formalin fixed, paraffin embedded and probed with an antibody against Erbb2 as indicated. Representative images were taken at 200X magnification. (**D**) Following three days of culture, tumor tissues in triplicates were formalin fixed and paraffin embedded. Tumor slices treated with the vehicle control (first column), cetuximab (second column), trastuzumab (third column), cetuximab + trastuzumab (fourth column) and trastuzumab + MK0752 (fifth column) were stained for H&E (first row) and selectively probed with antibodies against Ki-67 (second row) and active caspase-3 (third row) as indicated. Representative images were taken at 200X magnification. (**E**) Scatter plot depicting S-Score for responders (green) and non-responders (red) to trastuzumab (inverted triangles), cetuximab + trastuzumab (triangles) and trastuzumab + MK0752 (squares) (*n* = 21). Samples with S-Score greater than 19.1 were categorized as responders.

The observation that Notch signaling was significantly deregulated in certain wt *KRAS* tumors prompted us to rationally co-target both these pathways (Notch and EGFR). To this end, we treated cetuximab resistant tumor samples (*n* = 21) with a Notch inhibitor (MK0752) either alone or in combination with cetuximab. However, none of the Notch deregulated tumors were sensitive either to MK0752 alone or a combination of MK0752 + cetuximab. Although there was a significant inhibition of the pharmacodynamic marker HES1 post-treatment with MK0752, no noticeable change was observed in tumor morphology, tumor cell proliferation and induction of apoptosis between the control and treatment arms (Fig. 4A). The predicted S-Score for all these samples was calculated and plotted to evaluate the response profile (Fig. 4B). GSEA data analysis revealed that in addition to Notch, Erbb2 was also deregulated in non-responder group. This prompted us to study the basal expression levels of Erbb2 across the subset of tumors non-responsive to MK0752 and cetuximab (Fig. 4C). All 21 samples had elevated levels of Erbb2 (2+ to 3+) which suggested that co-targeting Erbb2 along with the EGFR signaling axis could be a potential strategy to boost the antitumor response. In about 25% of Erbb2 over expressing tumors (5/21), trastuzumab in combination with cetuximab resulted in a moderate antitumor effect in terms of reduction in tumor morphology and tumor cell proliferation with concomitant activation of cleaved caspase-3 (Fig. 4D). The remaining samples did not respond to this particular combination. The S-Score for the specific treatment arms (i.e. trastuzumab and cetuximab + trastuzumab) was calculated and the segregated response profile was analyzed for understanding the potential benefit, if any (Fig. 4E). Since a significant increase in antitumor efficacy was not observed using these combinations we decided to explore the efficacy outcome in combination with Notch and Erbb2 inhibitors. A profound antitumor efficacy was observed in a majority of the tumors (16/21, ~76%) when they were treated with a dual combination of MK0752 and trastuzumab. There was an appreciable change in histology, tumor proliferation and induction of cell death in this combination arm compared with the control or other treatment arms (Fig. 4D and E), indicative of altered dependence on other oncogenic pathways that would further instigate the tumor cell proliferation, progression and survival. The response pattern was calculated as defined earlier (Fig. 4E)^26^. These data collectively indicate that Erbb2 along with the Notch signaling axis plays a key role in 75% of wt *KRAS* CRC (with respect to codons 12/13, 61 and 146) tumors and systematic delineation of tumor dependence on these pathways using a functional CANscript platform could aid crafting of combinatorial strategies for effective therapeutic interventions.

## Discussion

Colorectal cancer is a major cause of cancer related mortality in both developed and developing countries. Several pathways including peroxisome proliferator activated receptor (PPAR)^33^, mitogen activated protein kinase (MAPK)^34, 35^ and EGFR^36^ are known to be deregulated in CRC. Aberration of the EGFR network is documented in 30–90% of advanced CRC^3^. In mCRC patients, anti-EGFR targeted therapy has markedly improved survival but not without limitations^36^. Targeted agents approved for mCRC are vascular endothelial growth factor (VEGF) inhibitors (bevacizumab, ramucirumab and aflibercept), regorafenib and EGFR inhibitors^37^. Though significant progress has been made towards identification of predictive and prognostic biomarkers, ascertaining the population most likely to benefit from such therapy remains a big challenge^38^. It is now well perceived that response and/or malignant progression of disease is regulated by complex multifaceted pathways. Therefore, analysis of any single or panel of biomarkers may not be the sole determinant to accurately predict clinical outcome^20^. Further, not all potentially actionable targets convincingly yield benefit to a particular therapy.

Towards this end, we extended the current findings on biomarkers to our study in CRC using the CANscript platform, which maintains tissue integrity and heterogeneity *ex-vivo* similar to the native tumor microenvironment. This platform phenotypically complements molecular profiling as it provides a functional assessment of tumor sensitivity to drug treatment while preserving the native tumor-stromal-immune compartments in their entirety^39^. Using this system, we segregated patient tumors as responders or non-responders based on their response outcome to cetuximab. Non-responders identified in this study remained insensitive to cetuximab even at higher doses indicative of inherent biological mechanisms that might contribute to *de novo* resistance to this form of therapy^40^. Based on the *KRAS* mutation status we found a rationale for non-response to cetuximab in only 10% (4/40) of the tumors as they harbored the G12V/C mutation. Clinical observation indicates that mutation in *KRAS* is an early event in colon tumorigenesis^41^ and ~10–40% patients with CRC, in the western population, carry these point mutations. The mutations are most commonly present in codons 12 (~95%) and 13, and less commonly in codons 61 and 146 of the target *KRAS* gene^42, 43^. Recent retrospective studies have shown that not all *KRAS* mutations are equal in their biological characteristics. Mutations in codon 12 render the patient insensitive to cetuximab whereas the effect of mutated codon 13 is still controversial^44^. A comprehensive assessment of *KRAS* has enabled the identification of novel mutations in exon 4 that have overlapping but not identical biological activities^45^. Moreover, with the advent of new technologies such as Locked Nucleic Acid PCR (LNA-PCR), which are more sensitive than the standard sequencing, there is a possibility of identification of novel mutations that might confer resistance to anti-EGFR agents^46, 47^. In this line of clinical observation, a lesser proportion of patients harboring wt *KRAS* tumors are sensitive to EGFR inhibitors^15^; and a large proportion of the wt *KRAS* tumor samples in this study (27/36; 75%) did not exhibit sensitivity to cetuximab. Studies have shown that other factors, such as activating mutations in the RAS-RAF-MAPK or PI3K-AKT-mTOR signaling, that might as a consequence of oncogenic addiction switch; additionally confer resistance to anti-EGFR treatment^48^. Two candidate genes from these pathways, *BRAF* and *PIK3CA* are implicated in resistance to cetuximab in CRC. Though the negative prognostic value of mutated *BRAF*, similar to *KRAS*, is well established, it has been observed that the efficacy of anti-EGFR therapy in such patients is still debated^49^. Furthermore, mutations in exon 9 and/or 20 of *PIK3CA* are known to be associated with clinical resistance to anti-EGFR therapy. Concurrent with previous findings, that highlight the general low frequency of *BRAF* and *PIK3CA* mutations (compared to *KRAS*), we did not observe any mutations in either of these genes^50^. Furthermore, mutations in the EGFR pathway account for only 2–5% of the mutations in majority of the targets in CRC, within which only a small population (15–20%) of patients actually benefit from anti EGFR based therapy at clinic^23^. Therefore, the absence of mutations in these crucial genes in a subset of non-responders suggested the presence of other driver mechanisms/defects potentially impacting response to cetuximab. Towards this end, we analyzed the mRNA expression levels of *AREG* and *EREG*, two EGFR ligands that are mitogenic stimulators, promoting tumor growth and survival by autocrine/paracrine loop mechanism and are known to impact response to cetuximab in CRC patients in conjunction with *KRAS* mutation status^21, 28^. In-line with recent findings, we observed a strong link between low levels of *AREG* and *EREG* expression in ~22% of the non-responders (6/27 tumors) and cetuximab insensitivity. However, these findings do not address the underlying cause of non-response in the remaining tumor subsets.

To further delineate other possible mechanisms that underpin this response, we performed RNA microarray followed by GSEA to identify key pathways that might be deregulated in non-responders in contrast with responders. Earlier studies have independently reported the link between cetuximab response prediction and *KRAS* gene signatures with poor score and activation strength^29, 30^. Although these data highlighted a differential pattern of perturbation of tumor cell proliferation, metabolic state, growth factor receptor and cell survival pathway networks between the responder and non-responder population, the question of whether any of these can serve as a predictive biomarker warranted further validation. We observed that the major pathway enriched in CRC samples with reference to EGFR signaling was Notch signaling (Fig. 2C). The cross talk between the EGFR and Notch pathways has been identified in several cancers including CRC, suggesting that possible combinations of MK0752 with cetuximab in these tumors might elicit antitumor response^51^. Another important node shown to be differentially regulated in non-responders is Erbb2. The oncogenic Erbb2 is involved in the development of various cancer types and its over-expression is associated with an earlier recurrence and shortened survival^52^. In recent years the therapeutic opportunity of targeting HER2 for CRC has gained impetus. Additionally, it has been observed that a small subset of CRC over-expresses Erbb2^53^. Erbb2 activating mutations, copy number and elevated level of hergulins in circulation have emerged as important factors attributing to the aberrant EGFR signaling and subsequent resistance to anti EGFR therapy. Dual intervention of HER2 by trastuzumab in addition to tyrosine kinase inhibitors showed tumor regression in the patient derived xenograft models. Additionally, this trend was evident for *KRAS/NRAS/BRAF/PIK3CA* wild-type background tumors^22, 23, 54–56^. Furthermore, selective intervention of HER2 or HER3 along with EGFR has demonstrated the potential benefit of tumor regression in preclinical gastrointestinal carcinoma^57^. In our study, combined inhibition of Erbb2 and EGFR did not considerably shift the response pattern; however, we have focused our attention on Notch and Erbb2 pathways because their perturbation is also known to bypass classical EGFR signaling mechanism, thus potentially conferring resistance to cetuximab^58, 59^. To address the role of Erbb2 and Notch in tumors insensitive to cetuximab in mCRC, we treated the tumors with trastuzumab (a fully humanized monoclonal antibody directed against Erbb2) alone, and MK0752 (an inhibitor of γ-secretase required for Notch pathway activation) alone or MK0752 in combination with cetuximab. None of the 21 samples were observed to be sensitive to MK0752 monotherapy or the dual combination of MK0752 and cetuximab, suggesting that there are feedback loops and other redundant pathways that bypass the blockade to mediate tumor survival and progression. In addition, none of the tumors exhibited response to trastuzumab as a single agent. However, a small portion of samples (5/21) that were not sensitive to monotherapy exhibited significantly improved response to the combined treatment of anti-EGFR and trastuzumab similar to a recent study reported by Sartore-Bianchi *et al*.^58^. Approximately 76% (16/21) of samples responded to the dual combination of trastuzumab and MK0752, suggesting a possible cross-talk or mechanistic link between the Notch and Erbb2 pathways. These results suggest vulnerability of CRC to dual inhibition of Notch and Erbb2, leading to potential success of this treatment strategy. Our phosphoproteomic profiles combined with a high level of Notch downstream protein (HES1) highlighted the coordinated interaction of Erbb2, HES1 and Abl. Abl is linked to the Notch induced invasive-metastatic phenotype in CRC via reciprocal activation of DAB1, a protein induced by the Notch signaling pathway^59^. Our previous study in head and neck cancer highlighted the existence of multiple deregulated pathways in non-responder tumors, suggesting the presence of more than one driver of mechanisms and therefore the need to assess multiple rationale therapeutics^27^. This indeed suggests that although techniques like microarray, RTK proteomics and enrichment analysis might hint at potentially deregulated pathways, further validation is warranted in a clinically relevant functional platform where the efficacy of targeted drugs could be measured in a complex and heterogenous tumor context. Our data indicate that the Notch/Erbb2 signaling pathway(s) would play a critical role in response to therapy, not only in wt but also *KRAS* mutated tumors that are otherwise insensitive to cetuximab. Therefore, a combined blockade of these pathways in a preclinical *ex-vivo* platform offers a useful strategy to identify a tailored treatment for mCRC by effectively predicting the efficacy outcome of prospective drugs in a personalized setting. Further, Russo *et al*.^60^ demonstrated the development of resistance to cetuximab or panitumumab therapy over a period of time through novel mutations in *MEK*1^K57T^ and *KRAS* ^Q61H^, suggesting the underlying molecular heterogeneity and genetic alterations in tumors that were refractory to multiple targeted therapies^60^. This explains the possible reason for a small subset of tumors (5/21, ~24%) not responding to any of the tested combinations. Other strategies, such as the ones targeting MEK/PI3K-mTOR and MEK/EGFR nodes, have been used by different groups for elucidating response in cetuximab-resistant CRC tumors^61, 62^. The Notch pathway has been implicated in CRC tumors resistant to bevacizumab (anti-VEGF) and regorafenib (inhibitor of multiple kinases including VEGFR, PDGF, TIE-2 and FGF), suggesting the cross-talk of Notch with other pathways for tumor cell survival and proliferation and serving as a potential driver triggering resistance to anti-EGFR and anti-VEGF therapy^63^. Although the exact mechanism in this cohort is not clear, activation, interaction and subsequent localization of the Notch intracellular domain induces transcription of Erbb2 target genes leading to co-activation of the PI3K/AKT pathway, further justifies the co-targeting of these two axes^64^. For CRC, large scale retrospective and prospective trials are warranted to firmly establish the role of Erbb2/Notch as a resistance biomarker in a functionally diverse microenvironment. It will be worthwhile to determine whether signaling from these pathways converge by assessing the effects of multiple pathway inhibition in CRC.

Understanding the impact of treatment-induced mutations on survival and response to therapy is a major challenge in enabling personalized therapy to a clinically defined target patient population^36, 60^. Collectively our observations indicate that while multiple signaling nodes might contribute to tumor progression and survival, a functional platform integrated with molecular characterization could provide a unique framework for the generation of predictive classifiers to achieve better patient stratification, prior to testing rational combinations of therapeutics at clinics. Collectively, this study shows that functional outcome based biology approach can address the complexity of the tumor microenvironment by understanding the dynamic nature of neoplasms in terms of response outcome in clinically aggressive cancers such as CRC, which are otherwise confirmed as wt for validated *KRAS* mutations. This approach has the potential to identify effective therapeutic approaches in the premise(s) of precision oncology.

## Materials and Methods

### Patients

Tumor biopsies or surgical samples (*n* = 40) from patients with locally advanced/metastatic CRC were obtained and transported in Lactate Ringer’s buffer at 4 °C to the laboratory. Informed consent was received prior to collection of clinical tissue specimens from the patients. The experimental study protocol was duly approved by the Institutional Review Board (IRB) of the respective hospitals and cancer study centers (Stanley Medical College, Chennai), and carried out in accordance with regional guidelines and standards. A summary of the demographic and TNM staging is listed in Fig. 1A.

### Drugs

cetuximab (Merck Serono; Lot no. 143886), trastuzumab (Roche; Lot no. B3435B01) and Notch inhibitor, (MK0752, Selleck; Lot no. S266001) for this study were procured and prepared appropriately.

### CANscript explant culture

For *ex-vivo* analysis of drug responses in tumor, the samples were sectioned into 200–400 μm slices using McIlwain tissue chopper (Tedpella)^65^. The tumor sections were maintained in triplicates in RPMI 1640 media supplemented with 20% Fetal Bovine Serum (FBS), Insulin-Transferrin-Selenium (ITS), 1X GlutaMAX and 1X Penicillin, Streptomycin and Amphotericin B (ThermoFisher Scientific) as previously described^27^. The cultures were maintained in 48 well tissue culture plates for 72 h and treated with vehicle control (DMSO) or cetuximab (2 µM), trastuzumab (0.82 µM), MK0752 (54 µM) as single agent or as combinations (cetuximab and trastuzumab; cetuximab and MK0752 and trastuzumab and MK0752). Media with drugs was changed every 24 h. The tumor explants were harvested at baseline time (T0) and after 3 days (T72); the samples from different time points were assessed for viability and subsequently fixed in 10% buffered formalin and paraffin-embedded for histopathological (H&E) and immunohistochemical (IHC) evaluation^26^. Every time the efficacy evaluation was carried out for various drug combinations, non-responders to cetuximab were used as a control to re-confirm their response to cetuximab.

### Viability assay

Tumor cell viability was assessed by Cell Counting Kit-8 (CCK-8) (Dojindo). Briefly, one-tenth of the volume of the CCK-8 solution was added to each well of the plate and incubated at 37 °C for 3 h in a 5% CO_2_ incubator under humidified condition. The absorbance was measured at 450 nm using a multimode microplate reader (Enspire, Perkin Elmer). Baseline samples (T0) were used as calibrators (1X) to normalize inter-sample variation in the absorbance readings. The results were expressed as a percentage of tumor cell viability or inhibition relative to vehicle controls.

### Immunohistochemical analysis

Changes in the frequency of cell proliferation or cell death and representative signaling network marker(s) prior to and after drug treatment were evaluated by specific proliferation/apoptosis markers using rabbit polyclonal Ki-67 (Vector Laboratories, 1:600 dilution) and rabbit polyclonal caspase-3 antibodies (Cell Signaling Technology, 1:600 dilution). Pharmacodynamic markers were assessed using antibodies against Erbb2 (mouse monoclonal, Biogenex, clone CB11) and HES1 (rabbit polyclonal, Abcam, 1:100 dilution). Initial antigen retrieval was conducted in a Vector^®^ Antigen Unmasking Solution (Citrate based, Vector Laboratories) by exposure to microwave heating for 30 min. Quenching of endogenous peroxidase was conducted by incubating the sections in 3% H_2_O_2_ for 15 min. Protein blocking was carried out at room temperature (RT) for 1 h with 10% normal goat serum. The subsequent incubation steps were followed by washing in Tris Buffered Saline (TBS). Sections were incubated with primary antibodies at the aforementioned conditions followed by incubation with HRP-conjugated secondary antibody (SignalStain^®^ Boost IHC Detection Reagent; Cell Signaling Technology) for 1 h at RT. Chromogenic development of signal was completed using 3,3′-diaminobenzidine (DAB Peroxidase Substrate Kit; Vector Laboratories). Tissues were counterstained with Hematoxylin. Scoring and calculation of drug induced inhibition of individual tumor explants were performed as described previously^66^.

### S-Score generation

Values from histology (morphology), tumor cell proliferation, cell death and viability were used as inputs in a machine learning algorithm as described earlier^26^. The algorithm finally generates a single score (S-Score) which has the potential of predicting clinical outcome to therapy. A value greater than 19.1 may clinically correspond with response to the drug(s) tested in CANscript, while a value lower or equal to 19.1 may indicate non-response.

### Western blot analysis

The isolation of protein was carried out following lysis of tumor tissues in Radio-immunoprecipitation assay (RIPA, Sigma) buffer in the presence of protease and phosphatase inhibitors (Sigma) and quantified by modified Lowry method using Bio-Rad’s DC Protein Assay Reagent. Protein samples were run under denaturing conditions in SDS-PAGE in the presence of a standard molecular weight marker. Proteins from the gel were transferred onto nitrocellulose membrane and incubated with primary (p-EGFR, PY1068, rabbit polyclonal, dilution 1:500, Cell Signaling Technology; EGFR, rabbit polyclonal, dilution 1:1000, Cell Signaling Technology; and αTubulin, Imgenex) and secondary antibodies (Anti-Rabbit-HRP conjugate, Cell Signaling Technology), and detected by a ECL documentation system (GE Healthcare).

### Mutation analysis

Genomic DNA was extracted from tumor tissues using a QIAamp DNA Micro Kit (Qiagen) and subjected to PCR using region-specific primers to detect the mutational status of *KRAS* (codon numbers 12/13, 61 and 146), *BRAF* (codon 600) and *PIK3CA* (exons 9 and 20) by sequencing. DNA fragment containing *KRAS* mutation hotspots were amplified with the intron-based primers listed in the Supplementary Table 1. Reactions (carried out in triplicates) contained 2.5 mM MgCl_2_, 0.2 mM dNTPs, 1 μM of each primer, and 0.5 U of PhusionTaq (ThermoFisher Scientific) in a total volume of 50 μl. Cell lines such as SW480 (mutated *KRAS*) and Caco2 (wt *KRAS*) served as controls in PCR and sequencing reactions. The PCR was carried out at 95 °C for 5 min, followed by 25 cycles at 95 °C for 30 sec; 60 °C for 30 sec and 72 °C for 30 sec with a final extension for 5 min. The PCR products were resolved on 1.5% agarose gels. The amplicons were excised and purified using a QIAquick gel extraction kit according to manufacturer’s protocol (Qiagen) and processed for Sanger sequencing.

### RNA analysis

RNA later (Ambion) stabilized core biopsy samples were lysed using a hand-held homogenizer (Thermo Scientific) according to the standard operating procedure. Total RNA was isolated from pulverized tissues (5 mg) using an RNeasy Micro kit (Qiagen), and was subsequently assessed for quality by nanodrop (Thermo Scientific Nanodrop 2000). In each case 250 ng of total RNA was reverse-transcribed using the High-Capacity cDNA Reverse Transcription kit (Applied Biosystems) according to the manufacturer’s protocol.

### Quantitative real-time PCR

Each PCR was carried out in 20 µl of a reaction mix, containing 10 µl of SYBR select master mix (Applied Biosystems), 200 nM primers (primer information provided in Supplementary Table 1) and 100 ng of cDNA. The amplified products were run on 1% agarose gel to verify the correct product size for specified genes, i.e., *AREG* (72 bp) and *EREG* (76 bp). The following PCR conditions were used: UDG activation at 50 °C for 2 min followed by AmpliTaq Fast DNA polymerase (ThermoFisher Scientific) activation at 95 °C for 2 min, denaturation at 95 °C for 15 sec, annealing at 60 °C for 15 sec and extension at 72 °C for 1 min. All cycle threshold (Ct) values were normalized using *GAPDH* as the control (house-keeping) gene.

### Microarray analysis

Tumor RNA (cRNA) microarray was carried out using the Agilent Sure Print G3 Human GE 8 × 60 K microarray system (Agilent Technologies: http://www.chem.agilent.com). For RNA microarray a RIN value above 7 was used as a cut off. Approximately 200 ng of RNA extracted from tumor samples was reverse transcribed to generate Cy3 labeled amplified cRNA and was profiled using the Agilent Kits and platform (Agilent Technologies: http://www.chem.agilent.com). Array data was normalized using Feature Extraction software and Agilent’s GeneSpring software. Data was expressed as fold differences (both for upregulated and downregulated genes) compared with the corresponding control. Fold change below 1.5 was considered as insignificant for further validation. A heat map was generated and relationship (similarity of genes) was elucidated among different tumors based on their response status. ANOVA analysis of normalized data was performed to distinguish the differentially expressed genes (*P* ≤ 0.05) between responders and non-responders.

### Gene set enrichment analysis (GSEA)

GSEA was performed as described previously^31, 32^. The dataset was converted from probe sets to gene symbols and analyzed using the Java GSEA package. The input gene set database was comprised of the curated gene sets (c2) of the Molecular Signature Database version2 (MSigDBv2) which includes Reactome gene sets (http://www.reactome.org). A false discovery rate (FDR) below 25% was considered for significant enrichment. Gene sets enriched in non-responder compared with responder classes were ranked by a normalized enrichment score (NES), and statistical significance was determined by permutation of the gene tags.

### Reverse phase phosphoproteomic array (RPPA)

PathScan RTK signaling antibody array kit (#7982, Cell Signaling Technology) was used to measure 28 receptor tyrosine kinases and 11 downstream signaling nodes. CRC tumor tissues sensitive and insensitive to cetuximab were studied for characterization at baseline. Tissue slices were extracted with 1X RIPA (cell lysis) buffer in the presence of protease and phosphatase inhibitors (Sigma). Protein estimation was done using Bio-Rad’s DC Protein Assay reagents. Tissue lysates were diluted with array diluent buffer and added to the well at a concentration of 0.5 mg/ml. Incubation was performed at RT at slow orbital shaker for 3 h. Primary and secondary antibodies were added per the manufacturer’s instructions and chemiluminescent signals were detected using Gel Doc XR+ System (Bio-Rad). Each spot was quantified by image analysis system (Multi Gauge, Fujifilm) for further analysis.

### Statistical analysis

Statistical analysis for cell viability, cell proliferation, induction of apoptosis and gene expression was carried out as per the standard procedure using ANOVA or student’s ‘*t*’ test. *P* value was calculated to determine significant differences. All graphs and statistics were performed using GraphPad Prism.

## Electronic supplementary material

Supplementary Data
