# Supplementary material for: Rationally co-targeting divergent pathways in KRAS wild-type colorectal cancers by CANscript technology reveals tumor dependence on Notch and Erbb2
Source: Sci Rep. 2017 May 4;7:1502. doi: 10.1038/s41598-017-01566-x (PMC5431418; doi:10.1038/s41598-017-01566-x)
Supplement: Supplementary file 1 — Supplementary Data [file 41598_2017_1566_MOESM1_ESM.doc]

**Rationally co-targeting divergent pathways in *KRAS* wild-type colorectal cancers by CANscript technology reveals tumor dependence on Notch and Erbb2**

Nilesh Brijwani**^1a^**, Misti Jain**^1*^,** Muthu Dhandapani^2*^, Farrah Zahed**^1^**, Pragnashree Mukhopadhyay^4^, Manjusha Biswas^4^, Deepak Khatri^2^, Vinod D Radhakrishna^3^, Biswanath Majumder**^4^**, Padhma Radhakrishnan**^5^** and Saravanan Thiyagarajan**^2, 3 #^**

Divisions of Molecular Profiling**^1^**, Cancer Biology**^2^**, Oncology Pharmacology**^3^**, and Molecular Pathology**^4^**, Mitra Biotech, Bangalore, Karnataka, 560099, India; Cancer Biology Division at Mitra Inc. Woburn, MA, USA^5^; a PhD scholar with Symbiosis International University (SIU), Lavale, Mulshi Taluka, Pune, Maharashtra, 412115, India^a^.

^*^Authors contributed equally

^#^Corresponding Author:

Saravanan Thiyagarajan, Ph.D.

Mitra Biotech Private Limited

Suite 202, Narayana Nethralaya

Narayana Health City Campus,

Hosur Road, Bangalore- 560 099, Karnataka, India

Telephone: 91-8066660760

E-mail: saravanan@mitrabiotech.com

**SUPPLEMENTARY FIGURES**


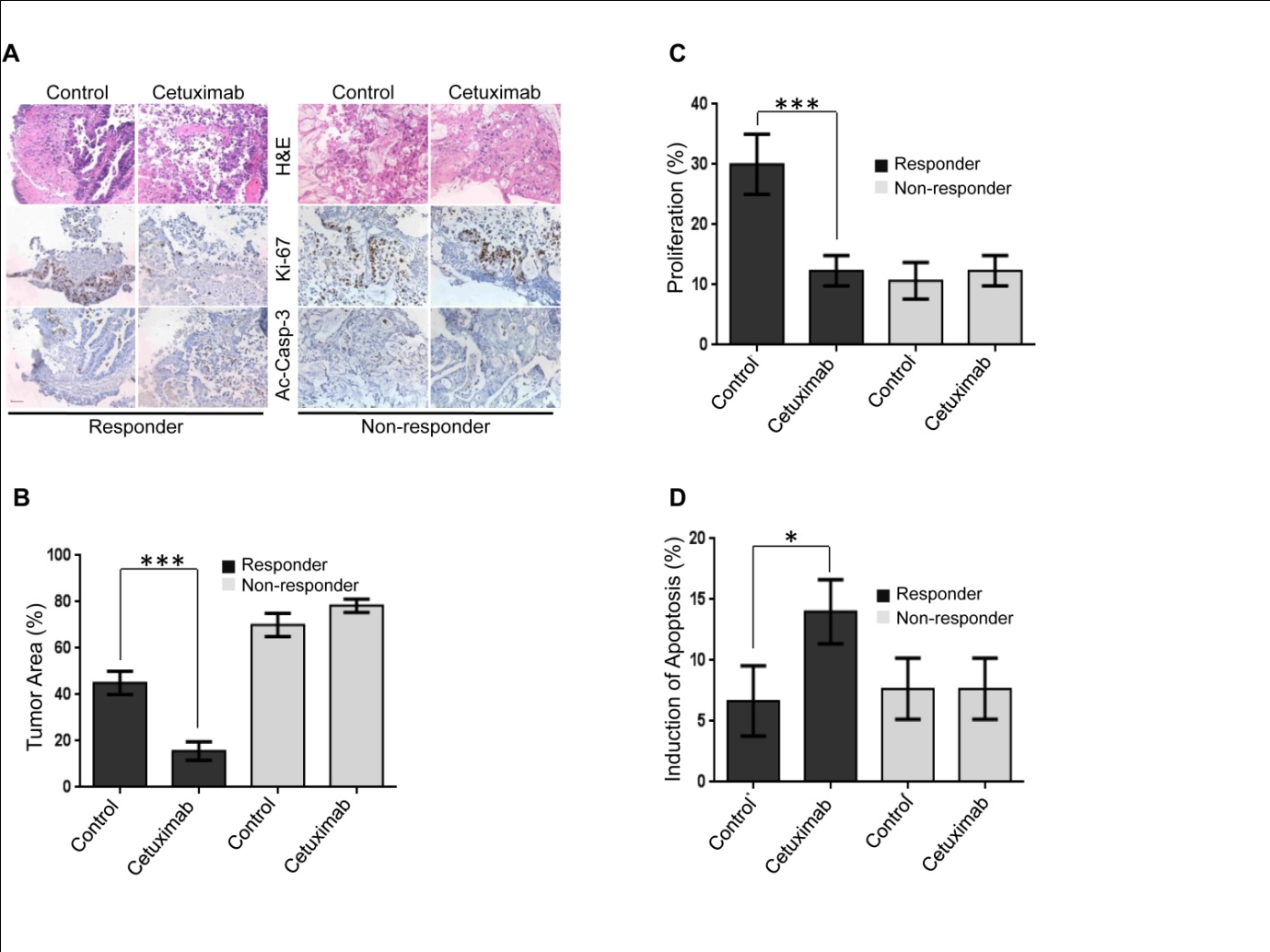


**Supplementary Fig. S1. Histological and immunohistochemical evaluation of CRC tumors sensitive and insensitive to cetuximab. A**, After three days of culture, tumor sections treated with cetuximab or vehicle control were fixed and embedded in paraffin. The representative images correspond to responders (left panel) and non-responders (right panel) to cetuximab therapy. The vehicle control (left column) and cetuximab treated (right column) tumor sections were stained with H&E (first row) and probed with antibodies against Ki-67 (second row) and active caspase-3 (third row). Image magnification, 200X. **B,** Quantification of representative H&E images depicting percent tumor area in responder (left panel) and non-responder (right panel) tumors to cetuximab. The bar diagram corresponds to vehicle control and cetuximab arms. Significance (****P* < 0.001, data for each treatment arm was captured in quadruplicate) was calculated by one-way ANOVA. **C,** Quantification of representative IHC images depicting percent tumor cell proliferation in responder (left panel) and non-responder (right panel) tumors to cetuximab. The bar diagram corresponds to vehicle control and cetuximab arms. Significance (****P* < 0.0004, data for each treatment arm was captured in quadruplicate) was calculated by one-way ANOVA. **D,** Quantification of representative IHC images depicting percent induction of apoptosis in responder (left panel) and non-responder (right panel) tumors to cetuximab. The bar diagram corresponds to vehicle control and cetuximab arms. Significance (**P* < 0.03, data for each treatment arm was captured in quadruplicate) was calculated by one-way ANOVA.


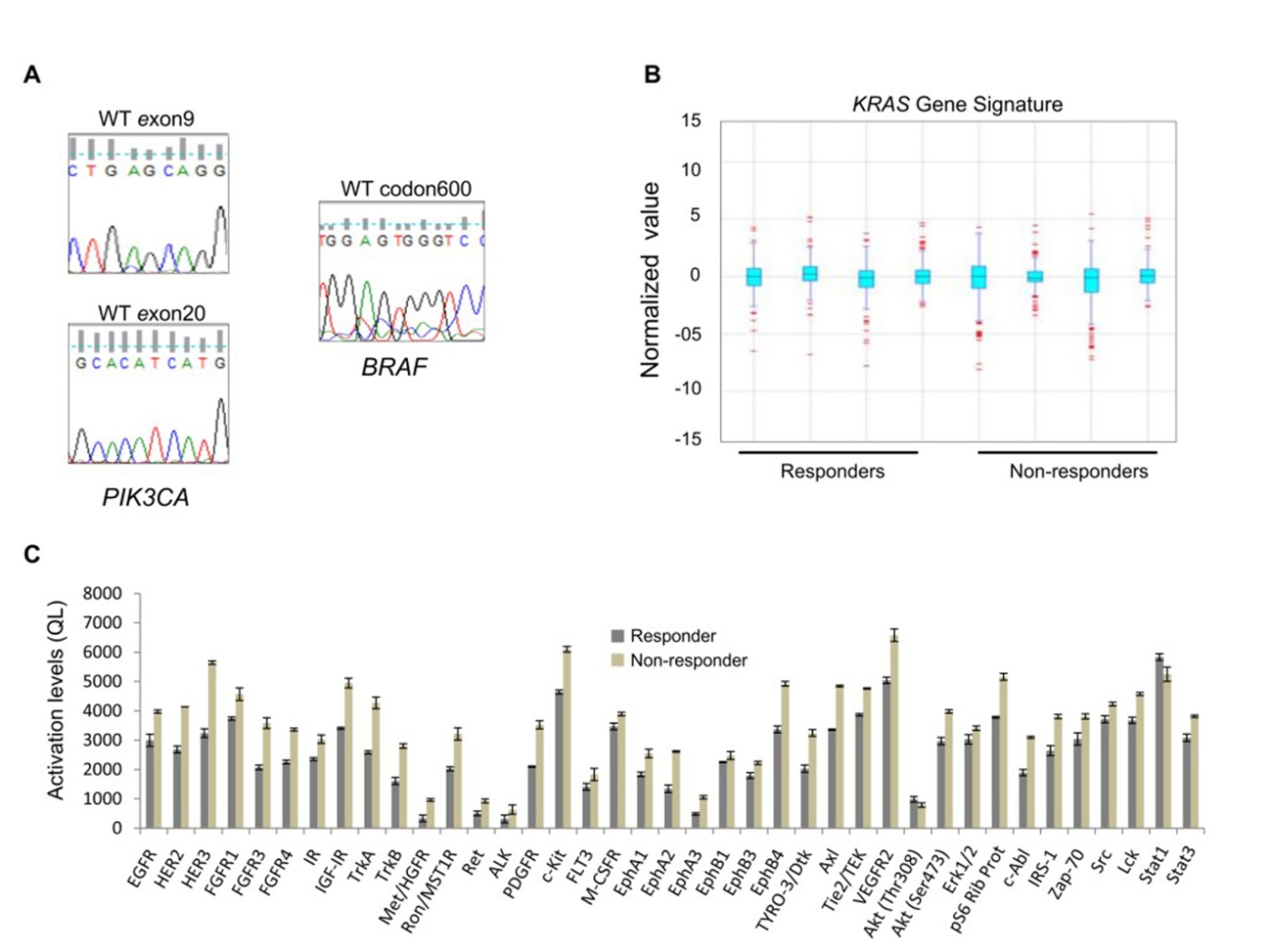


**Supplementary Fig. S2. Extended molecular profiling of wt *KRAS* tumors. A**, Genomic DNA was extracted from tumor tissues and subjected to PCR using region-specific primers to detect the mutational status of *BRAF* (codon 600) and *PIK3CA* (exon 9 and 20) by sequencing. Representative chromatograms for wild type *PIK3CA* (exon 9 and 20, left) and wild type *BRAF* (codon 600, right) are shown. **B**, Conservation of *KRAS* gene signature between responder and non-responder population. Box whisker plot for *KRAS* gene signature (147 genes) was generated using Genespring GX subsequent to microarray analysis (Agilent Sure Print G3 Human GE 8x60K Microarray platform) *n*=8. **C**, Intensity of each spot from RPPA array was assessed by densitometry and plotted graphically. Bar graph shows the relative activation levels (QL) of different pathways between responder and non responder population.


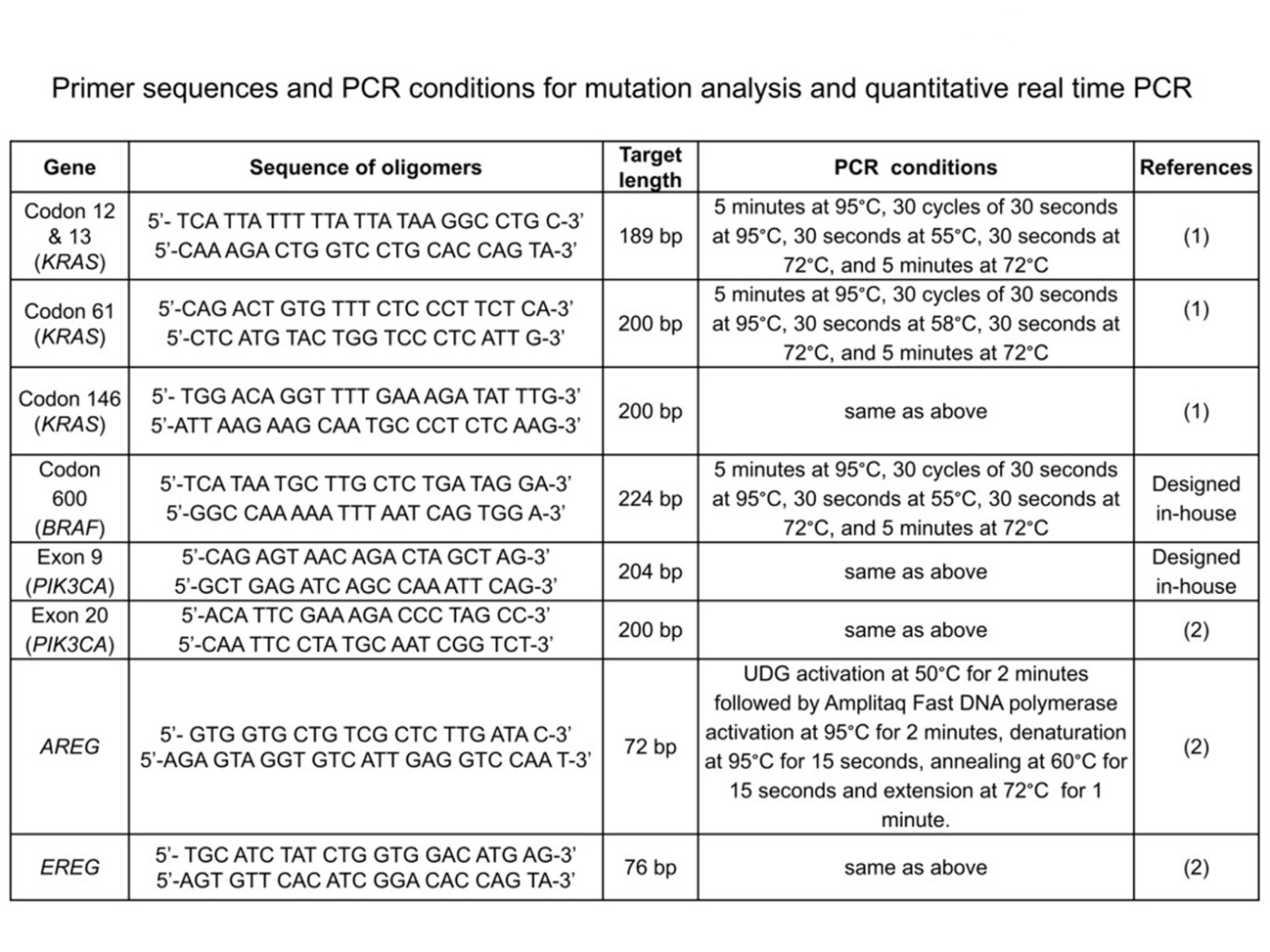


**Supplementary Table 1. Primer sequences and PCR conditions for mutation analysis and quantitative real time PCR.**

**Supplementary References:**

1. Tran PT, Shroff EH, Burns TF, Thiyagarajan S, Das ST, Zabuawala T, et al. Twist1 suppresses senescence programs and thereby accelerates and maintains mutant Kras-induced lung tumorigenesis. PLoS Genet. 2012;8:e1002650.

2. Saridaki Z, Tzardi M, Papadaki C, Sfakianaki M, Pega F, Kalikaki A, et al. Impact of KRAS, BRAF, PIK3CA mutations, PTEN, AREG, EREG expression and skin rash in >/= 2 line cetuximab-based therapy of colorectal cancer patients. PLoS One. 2011;6:e15980.
